# Rigorous science as the road to better public health

**DOI:** 10.1186/1478-7954-11-10

**Published:** 2013-07-10

**Authors:** Michael S Lauer

**Affiliations:** 1 Division of Cardiovascular Sciences, National Heart, Lung, and Blood Institute (NHLBI), 6701 Rockledge Drive, Room 8128, Bethesda, MD 20892, USA

**Keywords:** Public health, Population science, Obesity, Physical activity, Life-expectancy, Randomized trials

## Abstract

In the current issue of *Population Health Metrics*, two reports paint a bleak picture of American public health. Both physical inactivity and obesity remain highly prevalent; yet, it is not clear that increased physical activity will reduce the burden of obesity. There continue to be widespread disparities in life expectancy across United States counties. These reports appear against a backdrop of debate regarding how we should allocate our scarce resources for improving health: should we focus more on improving access to high-quality medical care, or should we instead focus on more and better public health interventions? While optimal solutions remain obscure, a look at prior successes suggests that ultimately they will come from the conduct and implementation of rigorous science, and in particular event-driven trials.

## 

Consider Alex Busko, who is about to start medical school. Recently he published in the *New York Times* a provocative letter about an impending doctor shortage. He suggested lowering bars for foreign-trained physicians [1]. Tim Wycoff, an experienced family physician suggested in reply that the United States focus more on public health interventions, “reining in the food and agriculture industry,” while promoting healthier diets along with increased physical activity [1]. Busko acknowledged that poor diet and lifestyle habits do account for much of our chronic disease burden, but “sadly I do not see this situation improving anytime soon [1].”

Just a few days after the *New York Times* published this dialogue, the “Look AHEAD” investigators published the results of a long-awaited trial of the impact of lifestyle interventions on cardiovascular outcomes in patients with diabetes [2]. The investigators randomized over 5,000 patients to an education control or to an intensive intervention of caloric restriction and increased physical activity. In some respects, the intervention worked: it led to greater weight loss and better physical fitness. But ultimately, it failed: there was no reduction in the rates of cardiovascular events or of total mortality.

So, maybe Busko was right to dismiss public health interventions. It’s simply impossible to improve diet and to increase physical activity. And even if we could, who’s to say that that will lead to better health?

In one of the two current reports, Dwyer-Lindgren et al. used BRFSS data to describe 10-year changes in the prevalence of obesity and insufficient physical activity across United States counties [3]. The authors found wide variations in both obesity and levels of physical activity. There was a weak correlation between changes in physical activity and obesity: for every 1% increase in physical activity, obesity prevalence was only 0.11% lower. The authors concluded that increasing physical activity alone might have little impact on the national obesity epidemic.

One reason we worry is that obesity may have an adverse impact on long-term improvements in life expectancy. Thus it is noteworthy that alongside Dwyer-Lindgren et al’s report, Wang et al. report using county-specific mortality data to describe changes in life expectancy between 1985 and 2010 [4]. Life expectancy has increased in both men and women with a lower gender gap; however, geographic variations in life expectancy persist, and in fact are worsening.

Taken together, these reports paint a bleak picture for United States public health. Obesity and physical inactivity remain highly prevalent, with gross geographic disparities. Increasing physical activity may not lessen obesity. Life expectancy may be improving, but we still lag behind much of the rest of the developed world, and we still suffer from gross disparities. To paraphrase Karl Malden, “What we will do?”

It is interesting that Wang et al. frame their investigation in the context of an American paradox: we spend more for health care than any other country, yet our health outcomes are relatively poor [5]. Why? Is it, as suggested by Busko[1], because we have too few doctors? Or because of inadequate access to health care? Or because, as suggested by Wycoff [1], Dwyer-Lindgren et al.[3], and the Look AHEAD investigators[2], public health interventions appear to have limited impact?

We cannot simply dismiss public health as ineffective. After all, some of the most powerful interventions we have come from public health: think public sanitation and vaccines. David Hemenway recently argued that public health is chronically underfunded for a variety of reasons [6]. The benefits of public health lie in the future, while people are oriented in the present. The beneficiaries and the benefactors in public health are often unknown; it’s difficult to get people excited about “statistical lives” as well as about scientists and public health officials who work in the background. And some public health interventions directly threaten the bottom line of powerful interests.

One might suggest another reason why public health interventions generate less excitement, at least among clinically oriented people whose primary focus is on clinical manifestations of disease rather than on surrogates like physical activity, body mass index, or blood pressure. Much, if not most, of the impressive reduction in cardiovascular death rates we’ve seen over the past 50 years stem from interventions that were identified through careful scientific investigations, including rigorous, large-scale randomized trials [7]. We identified the adverse impacts of elevated blood pressure and cholesterol levels through systematic epidemiological studies, studies that took into account possible biases and confounders [8]. We followed those studies with prospective randomized trials, which clearly demonstrated benefits [7].

A clinical investigator might ask for similar scientific rigor in the sphere of population health. Observational studies, like those published in the current issue of *Population Health Metrics,* should measure and account for confounders like income, education, smoking rates, pollution levels, health care spending, and public health policies. And, when possible, a clinical investigator might insist on performance of randomized trials before promulgation and implementation of new public health policies. We should have the humility to say that, right now, we do not know what “the right level” of physical activity is. Should we be recommending 150 minutes a week when 10 minutes a day may be adequate [9]? Should we be recommending lower calorie, low-fat diets when other kinds of diets may actually be better for preventing cardiovascular events [10]?

Some argue that it’s not possible to conduct randomized public health trials, trials that are analogous to those conceived by clinicians. Yet we have seen a number of examples in which randomized trials or “quasi-experiments” were successfully conducted within populations. Recently completed randomized trials assessed the health impacts of access to medical care[11], prescription drug coverage[12], and better quality housing [13]. A quasi-experiment suggested that mass screening for neuroblastoma did not reduce cancer mortality [14]. Esther Duflo has conducted numerous randomized trials testing the impact of economic policies on population well-being [15].

In our current environment of integrated health care and “big data,” it may be increasingly possible to conduct large-scale experiments at relatively low cost. Rather than arguing that randomized trials are not possible, we should instead focus our energies on how to conduct many trials, especially event-driven trials that will ultimately inform policies and practices that work. The National Institutes of Health Common Fund Health Care Systems “Collaboratory” is currently funding seven pragmatic trials that focus on a wide range of high-prevalence conditions, including suicide, back pain, hospital-acquired infections, hypertension, renal failure, and colon cancer, all as part of an effort to gain expertise in how to leverage large-scale information infrastructures to answer critical questions of relevance to public health quickly, robustly, and efficiently [16]. At the same time, the newly formed Patient-Centered Outcomes Research Institute (PCORI) is launching a major infrastructure effort that aims to create platforms upon which many “patient-centered” effectiveness studies could be designed and conducted in short order [17]. In an ideal world, diverse stakeholder groups, including clinical and public health practitioners and researchers, would come together, exploiting new opportunities afforded by big data, integrated health care, and when appropriate, one of science’s most powerful of tools, randomization [18].

We often hear that we cannot insist on doing trials to test absolutely everything, and, if we had, we never would have built sewer systems or implemented anti-smoking policies. Sanitation and smoking, though, are exceptions that prove the rule. In both cases, carefully conducted observational studies found huge effect sizes. In those rare cases where effect sizes are huge, randomized trials are not needed.

Alex Busko has launched his medical career on a positive note, asking provocative questions and inviting rigorous dialogue [1]. As is clear from the results of the Look AHEAD study [2] and the currently published reports of Dwyer-Lindgren et al. [3] and Wang et al. [4], we are a far way off from knowing what kinds of lifestyle and public health interventions, if any, might improve the poor health outcomes associated with the obesity epidemic. Yet we can also take comfort that through the promotion and conduct of rigorous observational and experimental science, it’s only a matter of time before we get there.

## Abbreviations

Look AHEAD: Look at action for health in diabetes; BRFSS: Behavioral risk factor surveillance system; NHANES: National health and nutrition examination survey; PCORI: Patient-centered outcomes research institute

## Competing interests

The authors declare that they have no competing interests. Dr. Lauer is a full-time federal employee and wrote this commentary as part of his official duties.

## Author’s Contributions

Dr. Lauer wrote this commentary and is its sole author.

## Author’s Information

Dr. Lauer is a cardiologist and a trained clinical epidemiologist. He is the Director of the NHLBI’s Division of Cardiovascular Sciences, which oversees a $1.5 billion portfolio of basic, translational, clinical, and population-based research.
